# Long and Short-term Metformin Consumption as a Potential Therapy to Prevent Complications of COVID-19

**DOI:** 10.34172/apb.2023.066

**Published:** 2022-07-02

**Authors:** Elnaz Shaseb, Saba Ghaffary, Alireza Garjani, Elnaz Zoghi, Nasrin Maleki Dizaji, Somaieh Soltani, Parvin Sarbakhsh, Mohammad Hossein Somi, Parya Valizadeh, Ali Taghizadieh, Masood Faghihdinevari, Mojtaba Varshochi, Behrooz Naghily, Zhinous Bayatmakoo, Parviz Saleh, Sepehr Taghizadeh, Mehdi Haghdoost, Hamid Owaysi, Fatemeh Ravanbakhsh Ghavghani, Mohammad Kazem Tarzamni, Rojin Moradi, Fateme Javan Ali Azar, Saeid Shabestari Khiabani, Ardavan Ghazanchaei, Sana Hamedani, Shahabeddin Hatefi

**Affiliations:** ^1^Department of Pharmacotherapy, Drug Applied Research Center, Tabriz University of Medical Sciences, Tabriz, Iran.; ^2^Faculty of Pharmacy, Tabriz University of Medical Sciences, Tabriz, Iran.; ^3^Hematology and Oncology Research Center, Tabriz University of Medical Sciences, Tabriz, Iran.; ^4^Faculty of Pharmacy, Tehran University of Medical Sciences, Tabriz, Iran.; ^5^Road Traffic Injury Research Center, Tabriz University of Medical Sciences, Tabriz, Iran.; ^6^Liver and Gastrointestinal Disease Research Center, Tabriz University of Medical Sciences, Tabriz, Iran.; ^7^School of Medicine, Tehran University of Medical Sciences, Tehran, Iran.; ^8^Tuberculosis and Lung Diseases Research Center, Tabriz University of Medical Sciences, Tabriz, Iran.; ^9^Infectious and Tropical Diseases Research Center, Tabriz University of Medical Sciences, Tabriz, Iran.; ^10^Department of Radiology, Medical Radiation Sciences Research Group, Imam Reza Hospital, Tabriz University of Medical Sciences, Tabriz, Iran.; ^11^Faculty of Medicine, Tabriz University of Medical Sciences, Tabriz, Iran.; ^12^Imam Reza Hospital, Tabriz University of Medical Sciences, Tabriz, Iran.

**Keywords:** COVID-19, Diabetes mellitus, Metformin, Intubation

## Abstract

**Purpose:**

The aim of the study is to evaluate the effect of metformin in complication improvement of hospitalized patients with COVID-19.

**Methods:**

This was a randomized clinical trial that involved 189 patients with confirmed COVID-19 infection. Patients in the intervention group received metformin-500 mg twice daily. Patients who received metformin before admission were excluded from the control group. Patients who were discharged before taking at least 2000 mg of metformin were excluded from the study. Primary outcomes were vital signs, need for ICU admission, need for intubation, and mortality.

**Results:**

Data showed that patients with diabetes with previous metformin in their regimen had lower percentages of ICU admission and death in comparison with patients without diabetes (11.3% vs. 26.1% (*P*=0.014) and 4.9% vs. 23.9% (*P*≤0.001), respectively). Admission time characteristics were the same for both groups except for diabetes and hyperlipidemia, which were significantly different between the two groups. Observations of naproxen consumption on endpoints, duration of hospitalization, and the levels of spO_2_ did not show any significant differences between the intervention and the control group. The adjusted OR for intubation in the intervention group versus the control group was 0.21 [95% CI, 0.04-0.99 (*P*=0.047)].

**Conclusion:**

In this trial, metformin consumption had no effect on mortality and ICU admission rates in non-diabetic patients. However, metformin improved COVID-19 complications in diabetic patients who had been receiving metformin prior to COVID-19 infection, and it significantly lowered the intubation rates.

## Introduction

 Novel coronavirus infection was first reported in December 2019 in China, which led to a global viral pandemic. Some of the patients with COVID-19 develop ARDS characterized by ground glass changes on imaging and pro-inflammatory cytokine storm.^1-3^

 Epidemiological findings demonstrate several risk factors related to poor prognoses in patients with COVID-19. Known risk factors are obesity, hypertension, diabetes mellitus (DM), chronic kidney disease (CKD), and cardiovascular disease.^4^

 Besides lowering blood glucose, metformin has recently been shown to have a wide range of therapeutic effects.^5^ For instance, metformin has a therapeutic impact on viral and bacterial infections such as M. tuberculosis and L. pneumophila.^6,7^ Moreover, metformin lowers mortality rates in patients with sepsis.^8^ Furthermore, metformin can reduce the levels of certain inflammatory cytokines, including TNFα, which is related to pathways that are potential targets for pharmacotherapy in COVID-19.^9^

 We hypothesized that metformin would improve the outcome of hospitalized patients with COVID-19. However, Rajeshkumar et al reported fatal toxicity in mice after consuming a combination of hydroxychloroquine and metformin.^10^ In this study, we assessed all reported side effects of this combination in humans, as well as the role of metformin in COVID-19 patients.

## Materials and Methods

###  Study design

 In this randomized clinical trial, 189 patients (85 in metformin and 104 in control groups) who had laboratory or clinically-confirmed SARS-CoV-2 infection were included. Patients were recruited from COVID-19 wards of Imam Reza hospital in Tabriz, Iran, between March 20 and April 5, 2020. Patients who were directly admitted to the ICU were omitted. Patients who received metformin before admission were excluded from the control group. Demographic information, laboratory data, and inflammatory markers were recorded for all patients. Clinical symptoms such as vital signs, O2 saturation, fever, cough, and QT intervals were recorded for all patients.

 Computed tomographic (CT scan) images were recorded and considered in combination with clinical symptoms and PCR tests for COVID-19 diagnosis. Pregnant women, patients with type I diabetes, ketoacidosis, severe kidney failure (GFR < 30 ml/min), severe respiratory failure, retinopathy, G6PD deficiency, and children were excluded from the study. Furthermore, patients who were already taking metformin were excluded from the control group.

###  Intervention

 patients who were randomly (block randomization) selected for the intervention group received metformin, 500 mg twice daily. To assess the long-term effect of metformin, patients who had metformin in their drug regimen prior to COVID-19 infection were excluded from the control group but not from the intervention group. All patients received metformin during hospitalization in the intervention group. Patients who were discharged before taking at least 2000 mg of metformin were excluded from the study.

 All patients in both groups received local practice protocol for COVID-19. Patients in the control group did not receive any extra medication.

 The local practice protocol for COVID-19 between March 20 and April 5, 2020, was hydroxychloroquine, ribavirin, and a combination of lopinavir-ritonavir.

 Metformin was well tolerated, and no patient reported any specific side effects during administration.

###  Follow up

 Vital signs, need for ICU admission, need for intubation, and death rates were assessed during the hospitalization period as primary outcomes for all patients. In the intervention group, QT-interval prolongation was measured to monitor the drug interaction between hydroxychloroquine and metformin.

###  Statistical analysis

 Data were described as number (%) for categorical variables and mean (SD) or median (interquartile range) for continuous variables for each group. Normality assumption for continuous variables was checked visually and analytically by using a Q-Q plot and Shapiro-Wilk test. Two-tailed hypothesis tests were set to test the equality of the two groups at a significance level of 5%.

 Chi-square and Fisher’s exact tests were performed for the between-group comparisons of categorical variables. Furthermore, an independent t-test was used for the between-group comparison of continuous variables.

 In addition, we used a generalized linear model with identity, logit, and log link functions to assess the effect of the intervention on the continuous, count, and binary outcomes, respectively, adjusted for diabetes, HLP, and naproxen as confounders. SPSS version 26 was used for data analysis.

## Results and Discussion

**Figure 1:**
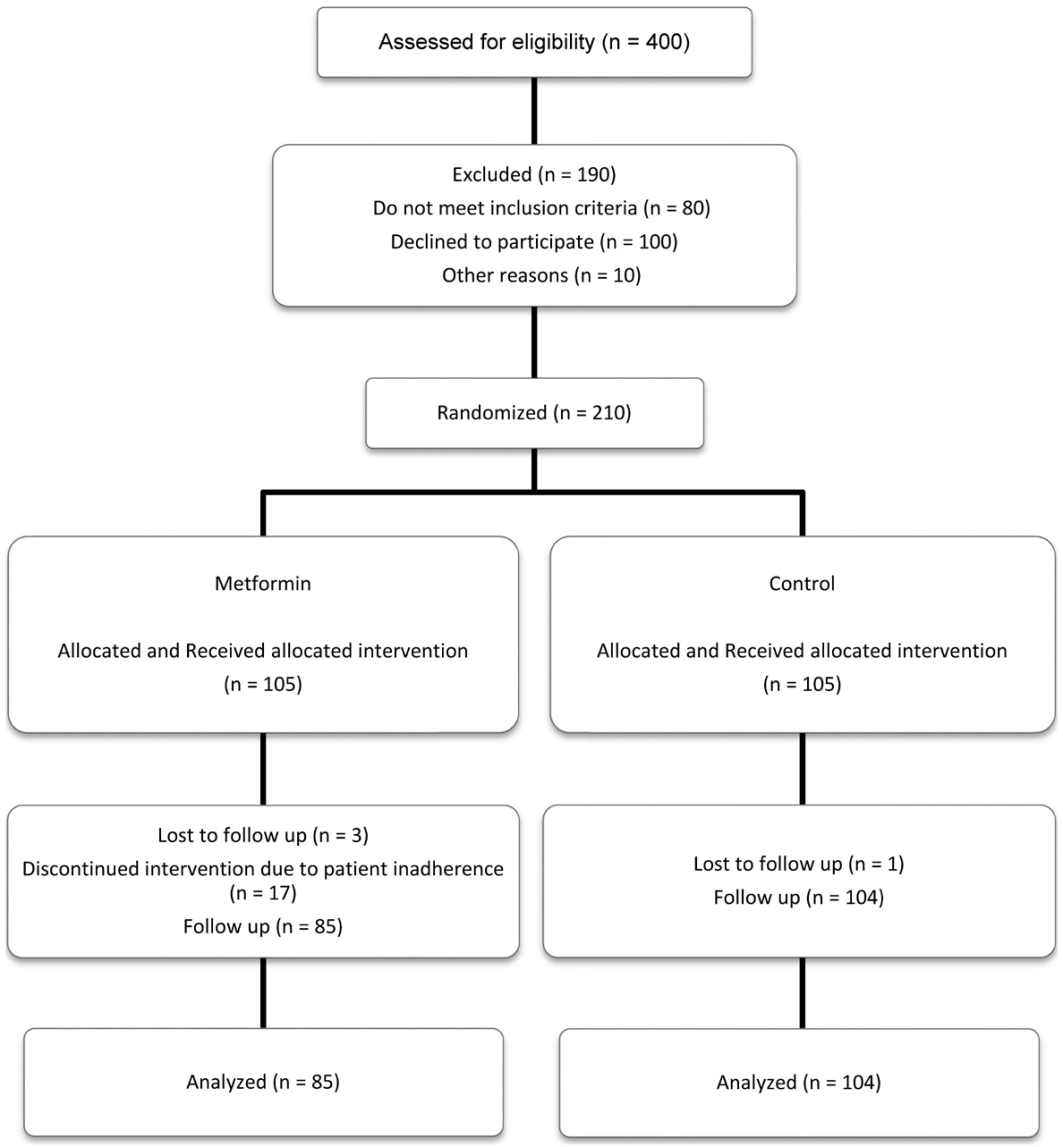
Screening, randomization, and follow up of study participants

 Four hundred patients were assessed for eligibility for the study, of whom 210 patients were eligible and were randomized to the intervention or control group. Twenty-one out of 210 patients withdrew from the study. Finally, 104 patients in the control group and 85 patients in the intervention group completed the study (Figure 1).

 Baseline characteristics of participants are presented in Table 1. Based on the results, coughing was the most common symptom among patients in both groups (76.5% in the intervention group and 77.9% in the control group).

 Comorbidities and medication-related information were also assessed. The number of patients with established diabetes and hyperlipidemia was not same between two groups (*P* = 0.001 and p = 0.02, respectively). Furthermore, the history of administered drugs before and during their admission was compared between the two groups. The intervention group has a significantly more frequent history of glibenclamide and atorvastatin usage in their drug history (*P* < 0.05) and a higher incidence of naproxen administration, and a lower incidence of IV antibiotics administration during their admission (*P* < 0.05). No significant difference was found in baseline values of imaging data (type, location, and percent of involvement) between the two groups. Diabetic patients who had been receiving metformin prior to COVID-19 infection had a lower need for ICU admission and mortality rate compared to non-diabetic patients (11.3% vs. 26.1% (*P* = 0.014) and 4.9% vs. 23.9% (*P* ≤ 0.001), respectively).

**Table 1:** Demographic and clinical characteristics of the patients

**Characteristics**		**Intervention (n=85)**	**Control (n=104)**	***P*** ** value**
Demographic	Age (y)	55.75 ± 17.99	59.00 ± 15.98	0.190
	Gender-male (%)	42 (49.4%)	61 (58.7%)	0.204
	BMI	28.79 ± 5.27	28.25 ± 4.64	0.509
	Smoking	2 (3.2%)	4 (4.9%)	0.599
	Opioids	1 (1.6%)	1 (1.2%)	0.849
Confirm PCR	Positive	54 (63.5%)	62 (60.2%)	0.640
Vital sign	SPO2	89.92 ± 5.24	89.31 ± 4.69	0.401
	Respiratory rate	23.46 ± 10.55	23.51 ± 10.01	0.971
	Pulse rate	87.34 ± 16.90	88.33 ± 14.91	0.670
	SBP	120.46 ± 14.67	122.03 ± 14.11	0.456
	DBP	77.15 ± 11.40	77.25 ± 7.72	0.946
	Temperature	37.28 ± 0.71	37.29 ± 0.69	0.984
Laboratory findings	WBC	6111.76 ± 252.68	6553.4 ± 369.82	0.350
	Neutrophil	75.02 ± 13.62	77.78 ± 9.15	0.122
	Lymphocyte	22.75 ± 12.73	20.00 ± 8.73	0.085
	Hemoglobin	13.32 ± 1.72	13.20 ± 1.85	0.649
	Platelet	354.50 ± 13.01	281.23 ± 104.69	0.674
	Creatinine	1.02 ± 0.01	2.42 ± 0.02	0.355
	AST	35.38 ± 2.63	56.87 ± 10.32	0.158
	ALT	27.46 ± 5.98	135.93 ± 33.65	0.359
	LDH	538.70 ± 19.46	596.45 ± 28.55	0.131
	CPK	151.44 ± 27.33	195.88 ± 16.18	0.123
	Sodium	136.40 ± 3.40	135.57 ± 3.00	0.078
	Potassium	4.11 ± 0.47	4.33 ± 3.47	0.564
	Magnesium	1.97 ± 0.29	1.94 ± 0.34	0.490
	PT	13.58 ± 1.62	14.07 ± 1.64	0.057
	PTT	34.87 ± 7.52	34.68 ± 10.12	0.890
	INR	1.04 ± 0.11	1.06 ± 0.10	0.293
Inflammatory markers	ESR	39.8 ± 25.49	42.70 ± 25.82	0.341
	CRP *(Ordinal)*	1.83 ± 1.13	2.00 ± 2.09	0.511
Symptoms	Fever	44 (51.8%)	53 (51.0%)	0.912
	Cough	65 (76.5%)	81 (77.9%)	0.818
	Dyspnea	50 (58.8%)	73 (70.2%)	0.103
	Myalgia	51 (60.0%)	49 (47.1%)	0.078
	Diarrhea	7 (8.2%)	12 (11.5%)	0.453
	Abdominal pain	7 (8.2%)	3 (2.9%)	0.102
	Headache	14 (16.5%)	14 (13.5%)	0.562
	Nausea	21 (24.7%)	18 (17.3%)	0.235
	Loss of appetite	15 (17.6%)	11 (10.6%)	0.160
	Weakness	21 (24.7%)	26 (25%)	0.963
	Shivering	16 (18.8%)	19 (18.3%)	0.922
Sensory complication	Taste	27 (43.5%)	34 (43.0%)	0.952
	Smell	28 (45.2%)	39 (49.4%)	0.620
	Hearing	1 (1.7%)	10 (12.8%)	0.016

BMI: Body Mass Index (kg/m^2^); PCR: polymerase chain reaction; SPO2: oxygen saturation; SBP: systolic blood pressure(mg/dL); DBP: diastolic blood pressure (mg/dL); WBC: white blood cell; ALT: alanine aminotransferase (units/L); AST: aspartate aminotransferase (units/L); LDH: lactate dehydrogenase; CPK: creatine phosphokinase.

 Besides, the need for intubation was higher in diabetic patients compared to non-diabetic patients (19.4% vs. 4.2%, *P* = 0.001). Modified marginal means of hospitalization time and spO2 level were recorded to reflect the impact of substantial differences in baseline values of diabetes, hyperlipidemia, and the amount of naproxen as an administered drug during hospitalization on the trial’s endpoints (Table 2).

**Table 2:** Comparison of adjusted marginal means of hospitalization duration and the level of spO_2_

**Variable**	**Intervention**	**Control**	***P*** ** value**
Hospitalization duration	5.70 (0.27)	6.00 (0.25)	0.52
SPO2 at discharge	90.54 (0.77)	91.75 (0.69)	0.13

SPO2: oxygen saturation

 Accordingly, no significant difference was observed in these parameters between the two groups. In the control group and the intervention group, the adjusted OR for intubation was 0.21 [95% CI, 0.04-0.99 (*P* = 0.047)]. after adjusting for diabetes, hyperlipidemia, and the amount of naproxen as an administered drug during hospitalization (Table 3). No significant difference was noted in QT-interval prolongation as a serious Drug-Drug interaction between groups at baseline (*P* = 0.92) and at discharge (*P* = 0.40).

**Table 3:** The Adjusted OR for intubation, fate, and intubation in the control group versus the intervention group

**Variable**	**OR**	**95% CI for OR**	***P*** ** value**
ICU admission	0.37	(.13,1.09)	0.073
Fate	0.26	(.06,1.06)	0.060
Intubation	0.21	(.04,.99)	0.048

ICU: intensive care unit.

 According to these results, metformin was significantly effective in reducing the need for invasive mechanical ventilation. However, metformin did not reduce the need for ICU admission, mortality rate, or length of hospital stay.

 Some observational studies have revealed that previous metformin use in diabetic patients is associated with a significantly reduced COVID-19 mortality. The latest meta-analysis of these studies was conducted by Kow and Hasan, which reported an overall significantly decreased mortality (OR = 0.62, [95% CI: 0.43–0.89]).^11^

 In an analysis by Bramante et al. on electronic health records (EHR) of 9,555 obese patients (BMI > 25 kg/m^2^), it was revealed that COVID-19 mortality was significantly lower in patients who had a history of previous metformin use (OR 0.32 [95% CI: 0.15- 0.66, *P* = 0.002]).^12^

 Regarding the results of previous observational studies with a large sample and our study, it can be concluded that the history of prior metformin use with an indication (diabetes or obesity) has a more tangible benefit in preventing COVID-19 complications.

 In the most prominent recent cohort study analyzing data from 27,493 patients with diabetes registered in the South Korean National Health Insurance Service (NHIS) database, as in our study, no significant reduction in COVID-19 mortality was observed with metformin use. However, previous use of metformin was associated with a 12% lower incidence of COVID-19 in patients with diabetes, and metformin demonstrated a preventive effect.^13^ The limitation of observational studies is that they do not have accurate information about patients’ compliance and whether metformin is continued or stopped during hospitalization. In line with our study, a lower risk of ARDS (adjusted OR = 0.18 [95% CI: 0.05-0.62; *P* = 0.0070]) was shown in diabetic patients who received metformin during hospitalization.^14^

 In this study, we decided to run a subgroup analysis which revealed that previous use of metformin in diabetics was associated with a significant reduction in ICU admission rate and mortality compared to non-diabetics with no previous metformin use. Of course, Diabetes itself is a known risk factor for worsening COVID-19.^15,16^ Therefore, this finding is not related to the effects of metabolic characteristics related to diabetes and is associated with other less prominent effects of metformin; Potentially, the immunomodulatory effects may reduce the severity of the inflammatory response in infection in diabetic patients. It is necessary to take metformin for a long time to see its positive impact. This indicates that metformin can be used as a prophylaxis against the occurrence of severe complications of COVID-19. Further investigations are required to evaluate metformin’s role as a prophylactic agent for COVID-19 among the non-diabetic population.

 In diabetic population, the CORONADO (coronavirus SARS-CoV-2 and diabetes outcomes) multicenter study in France, which investigated the predictors of hospital discharge and 28-day mortality in diabetic patients, showed that younger age, routine use of metformin, and prolonged pre-hospital symptoms were accompanied with decreased mortality rates. Age-adjusted OR (95% CI) for metformin use as a favorable prognostic factor of discharge within 28 days was 1.46 (1.25, 1.71) (*P* value < 0.001).^17^

 They also conducted another study as an interim analysis on 2449 patients (of whom 1469 were already taking metformin, 953 were not), which reported that previous metformin use was associated with a significant reduction in 7- and 28-day mortality.^18^

###  Safety endpoints

 In our trial, the claim of fatal toxicity of HCQ in combination with metformin was not substantiated. Further studies with the approved seven-day hydroxychloroquine dose are required to confirm this finding. No cases of QT prolongation (more than 60 ms change in QT interval or QTc ≥ 500 ms) were encountered in the study. However, this might be related to the low accumulation dose of hydroxychloroquine (400 mg) in our patients.

 According to our national protocol, one of the conditions for referring patients to the hospital is an oxygen saturation level of below 90% and the need for respiratory support. Therefore, most of the patients’ symptoms were respiratory, and the average level of oxygen saturation was less than 90%. Admission requirements in the COVID-19 ward include a definitive diagnosis based on a positive RT-PCR test or clinical suspicion plus positive findings on a CT scan of the lungs. Interestingly, the false-negative rate in the initial RT-PCR test in the present study was about 40%, which in the latest review study averaged 13% and up to 54%, false-negative PCR has been reported.^19^ Another study was conducted at the same treatment center – Imam Reza hospital – on CT scan findings of COVID-19 patients. The specificity of this method was reported to be higher than in previous studies (OR = 77, [95% CI: 73–81]). Results, similar to ours, predominantly revealed ground-glass opacities or peripheral consolidations.^20^

## Conclusion

 In conclusion, metformin has an effect on the course and the prognosis of COVID-19. Therefore, performing a well-designed trial to evaluate its clinical effects on COVID-19 was valuable. Although our clinical trial failed to demonstrate metformin’s benefit on mortality and rate of ICU admission, it was effective in reducing the need for mechanical ventilation significantly among hospitalized COVID-19 patients. However, metformin improves related complications in patients with diabetes who had been receiving metformin prior to COVID-19 infection.

## Limitations

 Because of COVID-19 restrictions in the first few months of the pandemic, the randomization had some technical problems and was not performed thoroughly. Patients receiving metformin prior to the study were not matched for metformin dose and duration. Diabetes and hyperlipidemia were not distributed the same in both groups. Another limitation of our study was the short follow-up period.

## Acknowledgments

 We would like to acknowledge all healthcare professionals of Imam Reza hospital for collaborating with this research during the COVID-19 outbreak.

## Competing Interests

 The authors declare no financial or non-financial conflict of interests.

## Ethical Approval

 Full informed consent was obtained from the participants. The study was conducted under the Research Ethics Committee (REC) of Tabriz University of Medical sciences guidelines of good clinical practice under the authorization of the Ministry of Health and Medical Education and the Helsinki Declaration. The presented data are part of the preliminary results of a clinical trial that is registered with the Iranian Registry of Clinical Trials, number IRCT20160310026998N10 and the ethics code of IR.TBZMED.REC.1398.1309.

## Funding

 We also appreciate the partial financial support of the current research by Tabriz University of Medical Sciences under grant numbers 65209 and 65208.
